# Differences in cardiac adaptation to exercise in male and female athletes assessed by noninvasive techniques: a state-of-the-art review

**DOI:** 10.1152/ajpheart.00756.2023

**Published:** 2024-02-23

**Authors:** Zofia Lasocka-Koriat, Zuzanna Lewicka-Potocka, Anna Kaleta-Duss, Anna Siekierzycka, Leszek Kalinowski, Ewa Lewicka, Alicja Dąbrowska-Kugacka

**Affiliations:** ^1^Department of Cardiology and Electrotherapy, Medical University of Gdańsk, Gdańsk, Poland; ^2^First Department of Cardiology, Medical University of Gdańsk, Gdańsk, Poland; ^3^Institute for Radiology, Cantonal Hospital Aarau, Aarau, Switzerland; ^4^Department of Medical Laboratory Diagnostics—Fahrenheit Biobank BBMRI.pl, Medical University of Gdańsk, Gdańsk, Poland; ^5^BioTechMed Centre/Department of Mechanics of Materials and Structures, Gdańsk University of Technology, Gdańsk, Poland

**Keywords:** athlete’s heart, cardiac biomarkers, echocardiography, electrocardiography, sex-related remodeling

## Abstract

Athlete’s heart is generally regarded as a physiological adaptation to regular training, with specific morphological and functional alterations in the cardiovascular system. Development of the noninvasive imaging techniques over the past several years enabled better assessment of cardiac remodeling in athletes, which may eventually mimic certain pathological conditions with the potential for sudden cardiac death, or disease progression. The current literature provides a compelling overview of the available methods that target the interrelation of prolonged exercise with cardiac structure and function. However, this data stems from scientific studies that included mostly male athletes. Despite the growing participation of females in competitive sport meetings, little is known about the long-term cardiac effects of repetitive training in this population. There are several factors—biochemical, physiological and psychological, that determine sex-dependent cardiac response. Herein, the aim of this review was to compare cardiac adaptation to endurance exercise in male and female athletes with the use of electrocardiographic, echocardiographic, and biochemical examination, to determine the sex-specific phenotypes, and to improve the healthcare providers’ awareness of cardiac remodeling in athletes. Finally, we discuss the possible exercise-induced alternations that should arouse suspicion of pathology and be further evaluated.

## INTRODUCTION

Long-term exercise training, as associated with increased demand for cardiac output, promotes changes in cardiac morphology and function called the “athlete’s heart” (1). For many years, participation in competitive sporting events was limited to male athletes. However, since the second half of the 20th century, the number of female athletes has steadily increased. In the first edition of “Berliner Volksmarathon” in 1974, 3.3% of women took part, whereas in 2018 this percentage increased to 30.2% (2). Indeed, in 2018, for the first time in history, more female than male endurance runners competed worldwide (50.24% vs. 49.76%) (3). As most studies on cardiac adaptation to training investigated male athletes, it is important to determine the sex-specific phenotypes to better distinguish physiological remodeling from pathological changes.

Prolonged periods of exercise followed by a cumulative increase in preload and afterload contribute to concentric cardiac remodeling in the form of left ventricular (LV) wall thickening to a greater extent in males than females (4). According to Finocchiaro et al. (5), who examined over 1,000 elite athletes, an average ventricular mass was higher in males than in females, whereas indexed ventricular dimensions were greater in females. Approximately 15% of male athletes engaged in endurance sports developed concentric hypertrophy compared with only 4% of females. In contrast, female athletes exhibited a higher prevalence of eccentric remodeling compared with males. Indeed, eccentric hypertrophy is a normal finding in females as opposed to concentric remodeling, which should arouse suspicion of pathology and be further evaluated. Although female athletes are smaller and have lower lean body mass, the discrepancies between sexes cannot be fully explained by the different body size. Normalization of LV mass for body surface area (BSA) or height did not change the pooled outcomes, and males continued to significantly exceed females (6).

There are several factors responsible for sex-specific cardiac response to exercise (Table 1). One of them is the hormonal profile and, consequently, cardiac protein synthesis (7). According to Handelsman et al. (8), prior to the onset of puberty, there are minimal differences in androgen levels between males and females. From puberty onward, males have higher circulating testosterone concentrations, exceeding 15- to 20-fold that of females at any age, and a higher density of androgen receptors. Genetic and immunohistochemical tests revealed that these receptors are present in cardiac myocytes from multiple species, including infant and adult humans of both sexes (9, 10). The two main bioactive androgens—testosterone and its metabolite dihydrotestosterone, cause hypertrophy by acting directly on cardiac muscle cells, increasing the incorporation of amino acids into protein (10). Antiandrogenic therapy has been shown to reverse pathological cardiac hypertrophy in murine models (11), demonstrating that the hypertrophic effect of the androgenic agents is mediated specifically by hormone binding to the androgen receptor.

**Table 1.** The determinants of sex-specific differences in cardiac adaptation to exercise

Sex	Hormonal Profile	Metabolic Processes	Neuroendocrine Response	Gene Expression
Male	↑ Testosterone levels induce androgen receptors on cardiac myocytes causing hypertrophy	↑ Carbohydrate and ↓ fat oxidation during exercise	↑ Sympathetic activity during exercise	↑ Renin-angiotensin system gene polymorphisms associated with left ventricular hypertrophy
Female	↑ Estrogen levels suppress hypertrophy and age-related degradation of cardiac myocytes	↑ Fat and ↓ carbohydrate oxidation during exercise	↑ Parasympathetic dominance and withdrawal of sympathetic response during exercise	↓ Renin-angiotensin system gene polymorphisms associated with left ventricular hypertrophy

↑ relatively greater response; ↓ relatively lower response.

Conversely, estrogen has a protective effect on cardiomyocytes, suppressing cardiac hypertrophy and combating age-related degradation (12). What is more, the higher levels of circulating estrogen in females contribute to differences in substrate oxidation during exercise (13). Females derive more energy from fatty acids, whereas men predominantly from carbohydrate oxidation (14). It might be explained by reproductive role of women and greater priority for carbohydrate conservation under conditions of increased energy demand. As concentrations of estrogen change throughout a woman’s lifetime, it is important to determine the actual female’s hormonal status, so as to properly interpret the response to exercise. Most of available research, along with this review, focuses on premenopausal women with highest estrogen levels and enhanced reliance upon the lipid oxidation. Menopause leads to a decline in plasma estrogen concentrations and, therefore, influences female athletic performance and substrate utilization profile.

Moreover, sex differences exist in the neuroendocrine response during training. In the study of Davis et al. (15), during exercise on cycle ergometer males had significantly greater sympathetic nervous system drive than females, as assessed by increased levels of catecholamines. In keeping with sympathetic activation, prolonged training was responsible for higher systolic and mean arterial pressures in men. Women, on the contrary, have greater parasympathetic dominance and lower sympathetic control of heart rate than men (16). The increased catecholaminergic response in male athletes following endurance exercise may result in greater reduction in β-receptor responsiveness and, consequently, greater reduction in LV systolic function compared with females (17). What is more, discrepancies exist in the autonomic regulation of exercise-induced lipid mobilization. In men exercise promotes both β- and α-receptors activation, whereas only β-receptors are activated in adipose tissue of women (18). As these are α-receptors that inhibit lipolysis (19), women may compensate for diminished sympathetic drive during exercise by greater lipolytic response. However, the increased percent body fat per se may also contribute to an enhanced lipolysis in women and cannot be overlooked. Future investigations should further explore the autonomic regulation in male and female athletes and provide its clinical implications.

As presented, male and female athletes exhibit many morphological and functional differences that influence exercise-induced cardiac remodeling. Therefore, in this review we will summarize the current knowledge on sex-related changes in cardiac adaptation to training, with the use of electrocardiography (ECG), echocardiography, and biochemical blood tests.

## ELECTROCARDIOGRAPHY

ECG is a useful screening technique in athletes that helps distinguish exercise-induced physiological remodeling from pathological changes. Over the years, standardized criteria for ECG interpretation in athletes have been introduced. Since 2010, when the European Society of Cardiology (ESC) recommendations for ECG interpretation in athletes were published (20), standards of ECG analysis have undergone a number of modifications aimed at improving accuracy and effectiveness in screening for cardiac pathologies. In 2012, an international group of experts in sports cardiology created “the Seattle Criteria” for ECG interpretation (21) based on the emerging research to help physicians detect ECG abnormalities, potentially associated with sudden cardiac death (SCD). Sheikh et al. (22) proposed further changes, adding a “borderline group” to “training-related” and “training-unrelated” factors. This update excluded several specific ECG patterns if present in isolation, significantly reducing the burden of false-positive ECGs in athletes. Finally, in 2017, an international consensus was reached and the current recommendations for ECG interpretation in athletes were published (23) (Table 2).

**Table 2.** Variants of electrocardiographic changes in athletes based on international criteria

Training-Related	Borderline	Training-Unrelated
• Sinus bradycardia or arrhythmia • 1° AV block • Mobitz Type I 2° AV block • Incomplete RBBB • Increased QRS voltage for LVH or RVH • Early repolarization pattern • Ectopic atrial or junctional rhythm • ST elevation with T wave inversion in V1–V4 in black athletes • T wave inversion in V1–V3 in athletes aged ≤16	• Left axis deviation • Right axis deviation • Left atrial enlargement • Right atrial enlargement • Complete RBBB	• T wave inversion • ST segment depression • Pathological Q-waves • Complete LBBB • Epsilon wave • QRS duration ≥ 140 ms • Ventricular pre-excitation • Brugada Type 1 pattern • Prolonged QT interval • Profound sinus bradycardia < 30 beats/min • PR interval ≥ 400 ms • Mobitz Type II 2° or 3° AV block • ≥ 2 PVCs per 10 s tracing • Atrial and ventricular arrhythmias

AV, atrioventricular; LBBB, left bundle branch block; LVH, left ventricular hypertrophy; PVC, premature ventricular contraction; RBBB, right bundle branch block; RVH, right ventricular hypertrophy.

The normal ECG findings that represent physiological adaptation to endurance exercise include sinus bradycardia, first-degree atrioventricular block (AVB), Mobitz type 1 second-degree AVB, incomplete right bundle branch block (RBBB), isolated QRS voltage criteria of left (LVH) or right ventricular hypertrophy (RVH), early repolarization, ectopic atrial/junctional rhythm, ST elevation with T wave inversion (TWI) in V1–V4 in black athletes, and TWI in V1–V3 in athletes aged < 16. Borderline variants, such as left or right axis deviation, left (LAE) or right atrial enlargement (RAE), and complete RBBB, if present in isolation require no further evaluation. However, two or more borderline ECG changes are considered pathological and may warrant additional investigation.

The abnormal ECG variants in athletes that always require further assessment to exclude the presence of cardiac disease include TWI in two or more contiguous leads in an anterior, lateral, inferolateral, or inferior territory, ST segment depression, pathological Q-waves, complete left bundle branch block (LBBB), Epsilon wave, profound nonspecific intraventricular conduction delay ≥ 140 ms, ventricular pre-excitation, Brugada Type 1 pattern, prolonged QTc, profound sinus bradycardia < 30 beats/min, profound first-degree AVB ≥ 400 ms, advanced second- or third-degree AVB, multiple premature ventricular contractions, atrial and ventricular arrhythmias.

**Figure 1.**
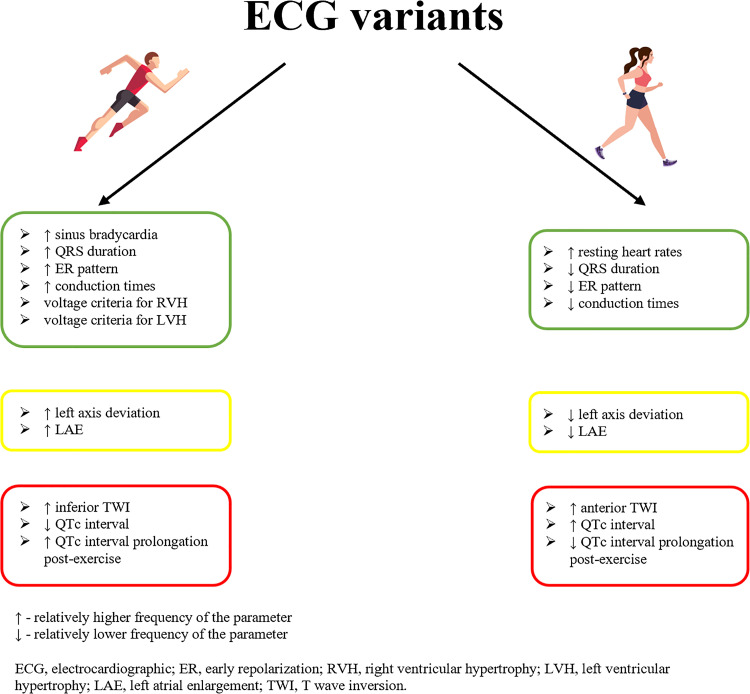
Comparison of electrocardiographic changes in male and female athletes.

There are several factors, such as age, ethnicity, sex, type of sport, and training duration, that influence the ECG interpretation in athletes. As previously mentioned, male and female athletes exhibit different cardiac adaptation to training and, therefore, present various exercise-induced ECG abnormalities. However, the international recommendations for ECG interpretation (23) include the same normal range values for both sexes, except from QTc cut-off values, which have been established at the level of 470 ms in male and 480 ms in female athletes. As most of available studies on ECG abnormalities concern male athletes with few data on female counterparts (24), we present a brief summary of sex-specific ECG differences in athletes (Fig. 1).

According to Pelliccia et al. (25), normal ECG pattern was more common among female athletes compared with males (78% vs. 55%; *P* < 0.01), and males had significantly larger proportion of either distinctly (17% vs. 8%; *P* < 0.001) or mildly abnormal (28% vs. 14%; *P* < 0.001) ECGs compared with females. However, research conducted with the application of Seattle criteria showed no statistically significant differences in both normal and abnormal ECGs between male and female athletes, but males were more likely to have two or more of training-related findings than females (85% vs. 59%, *P* < 0.001) (26). This discrepancy was largely explained by differences in the proposed definitions of TWI, QTc interval, and LAE between current Seattle criteria and the previous ESC recommendations from 2010.

Taking training-related changes into consideration, Bessem et al. (27), in his study on 1,436 athletes, showed that male athletes had significantly higher incidence of sinus bradycardia (38.3% vs. 23.0%), incomplete RBBB (15.0% vs. 3.7%), early repolarization (4.5% vs. 1.0%), isolated QRS voltage criteria for LVH (26.3% vs. 4.6%), had longer PR intervals (149 ms vs. 141 ms) and QRS duration (98 ms vs. 88 ms). In contrast, female athletes had significantly higher resting heart rates (69 beats/min vs. 64 beats/min). These findings are in agreement with previous studies by Mandic et al. (28) and by D’Ascenzi et al. (29), both demonstrating lower prevalence of atrioventricular and intraventricular conduction delays along with ECG criteria of LVH and RVH in females. However, Corîci et al. (30) found no significant sex-related differences in PR interval duration, QRS axis, incomplete RBBB, or early repolarization incidence. Although in the study of Finocchiaro et al. (5) QRS duration was similar between sexes (82 ± 12 ms vs. 87 ± 13 ms in females and males respectively; *P* = 0.156), female athletes very rarely exhibited the prolongation of this parameter >100 ms (3% vs. 33%; *P* < 0.001), which might be related to the different adaptation process between sexes, and lower incidence of concentric LV remodeling in women, as described further.

Among borderline variants, QRS frontal plane axis was more horizontal in the female group (63.2° vs. 66.4°; *P* = 0.019), in a study on more than 1,000 students of physical education and sport (31). There are conflicting results concerning the influence of sex on the incidence of LAE in athletes. Our recent research on amateur marathon runners showed that LAE was the most frequent borderline finding and occurred significantly more often in females than in males (48% vs. 20%; *P* = 0.03) (32). While Finocchiaro et al. (5) demonstrated that the presence of LAE was slightly higher in male athletes, not reaching the statistical significance (5.0% in males vs. 2.5% in females; *P* = 0.06). This discrepancy might be explained by other factors that impact exercise-induced cardiac response, such as type of sport, training volume, and years of practice. On the contrary, there were no remarkable differences in the incidence of RAE between male and female athletes (43% vs. 48%; *P* = 0.837) (32). However, when we assessed the acute changes, a significantly greater number of athletes of both sexes met the ECG criteria for RAE after the marathon run compared with baseline, with similar rate of LAE between the stages (32). These outcomes corresponded with our further echocardiographic study on the same population restricted to female amateur runners, in which a remarkable post-race increase in right atrial (RA) size and no relevant decrease in left atrial (LA) dimensions were noticed (33).

When analyzing the repolarization disorders, TWI in the anterior precordial leads was more prevalent in female athletes than in males (39.9% in females vs. 29.4% in males; *P* = 0.05) (29), whereas males had a higher incidence of inferior TWI (31.5% in males vs. 9.2% in females; *P* = 0.02). These findings are consistent with the previous study by Malhotra et al. (34), who detected anterior TWI in 338 individuals (2.3%), more frequently in females than in males (4.3% vs. 1.4%; *P* < 0.0001) and more commonly among athletes than in nonathletes (3.5% vs. 2.0%; *P* < 0.0001). The TWI was predominantly confined to V1 to V2 (77% of cases), and if extended to V3–V4, was more common in females. Because anterior TWI beyond V2, especially when preceded by J-point elevation, may mimic ventricular involvement in the form of hypertrophic cardiomyopathy (HCM) or arrhythmogenic right ventricular cardiomyopathy (ARVC) (35), it requires further investigation to exclude cardiac disease. However, such ECG abnormality is detected extremely rarely, usually restricted to singular leads, and should be considered a physiological variant in asymptomatic white individuals without a relevant family history.

Finally, female athletes in the postpubertal phase had significantly longer QTc intervals than males, owing to a longer ventricular action potential, as reported by Bessem et al. (27) and Corîci et al. (30). Our research, concerning postexercise changes in ECG parameters in male and female marathon runners, demonstrated significant QTc interval prolongation after endurance exercise in both sexes, however, to a higher degree in males (*P* = 0.029 for the interaction stage and sex) (32). This finding was supported by Ogedengbe et al. (36), who performed stress test by bicycle ergometer on 40 healthy individuals and presented a larger increase in the QTc duration in males compared with females (*P* < 0.05). Since prolonged QTc interval is an independent risk factor of SCD due to malignant arrhythmias (37), it is not surprising that the incidence of SCD is higher in males than in females (38). Corrado et al. (39) in their study on more than 110,000 athletes reported an incidence rate of SCD of 2.6/100,000 person-years in males compared with 1.1/100,000 person-years in females, whereas according to Marijon et al. (40) only 5.2% of exercise-related SCD occurred in women, which corresponded to male:female ratio of 18:1. Moreover, Finocchiaro et al. (41) showed that sudden arrhythmic death syndrome (SADS) was the most frequent cause of death (42%), in his research on 357 suddenly deceased athletes, with myocardial diseases responsible for only 35% of cases.

Considering the aforementioned data, the 12-lead ECG is a relatively simple and inexpensive method that strengthens the limited diagnostic efficacy of medical history and physical examination during preparticipation evaluation of athletes. Although most of the routinely performed ECGs are normal with only minor training-related changes, there are several ECG patterns principally recognized in endurance athletes of younger age and male sex that raise clinical suspicion of cardiovascular disease. These include increased precordial R or S wave voltages, deep Q waves, and interventricular conduction or repolarization disorders. If present, further echocardiographic assessment of cardiac chambers’ morphology and function is required, so as to early detect additional signs of underlying structural heart disease. Moreover, the application of 24-h Holter ECG monitoring would help identify potential arrhythmia triggers that account for the vast majority of sudden deaths in athletes.

In conclusion, both male and female athletes exhibit abnormalities in the ECG examination. This is why ECG screening in elite and nonelite athletes should always be considered, and ECG recommendations for athletes rather than for the general population should be used. As exercise-induced cardiac remodeling proceeds differently between sexes, it is important to implement sex-related cut-off values, which are not yet available, to increase the sensitivity and specificity of ECG interpretation, and to reduce the number of false-positive and false-negative results.

## ECHOCARDIOGRAPHIC EXAMINATION

Echocardiography is an easily approachable and rapidly developing method that enables accurate evaluation of cardiac remodeling in athletes. The introduction of new concepts, such as strain rate or three-dimensional (3-D) echocardiography, influenced current knowledge with further analysis of structural and functional changes in this population. The extent of cardiac remodeling varies across the sport disciplines, depending on the hemodynamic overload associated with training (42). Indeed, endurance athletes adapt primarily by increasing the size of ventricles and atria due to the largest increase in preload, whereas resistance athletes tend to present with concentric chamber hypertrophy. In this chapter, we will summarize existing research on exercise-induced echocardiographic changes in athletes, with an emphasis on sex-specific responses. The assessment of each cardiac chamber includes structural and functional adaptation to training with explanation of underlying physiological mechanisms. Tables 3 and 4 cover normative values for biventricular and biatrial echocardiographic parameters in male and female athletes, to better distinguish physiological from pathological range of measures. However, there is a lack of studies on 3-D phasic function of RA in male athletes. Therefore, values of these parameters were not included in Table 4.

**Table 3.** Normative values for biventricular morphology and function in athletes assessed by two- and three-dimensional echocardiography

First Author	No. of Athletes	Parameter	Male Athletes	Female Athletes
Finocchiaro et al. (5)	1,083 (59% males)	LVEDD, mm	54 ± 5	49 ± 4
		IVSd, mm	10.0 ± 1.3	8.7 ± 1.2
		PWd, mm	9.6 ± 1.2	8.4 ± 1.2
		RWT	0.36 ± 0.04	0.35 ± 0.04
		LVM index, g/m^2^	101 ± 21	83 ± 17
Roslan et al. (42)	100 (50% males)	LVEDV index, mL/m^2^	67.4 ± 14.8	57.3 ± 11.6
		LVESV index, mL/m^2^	27.8 ± 14.8	24.0 ± 7.6
		LVEF, %	60.5 ± 4.1	61.6 ± 4.0
		LV GLS, %	−20.0 ± 2.4	−21.9 ± 2.5
		LV GCS, %	−21.0 ± 2.8	−22.8 ± 2.4
Caselli et al. (43)	1,145 (61% males)	*E*-wave velocity, cm/s	84 ± 15	91 ± 14
		*A*-wave velocity, cm/s	46 ± 10	49 ± 10
		*E*/*A* ratio	1.92 ± 0.51	1.94 ± 0.49
		IVRT, ms	85 ± 14	80 ± 12
		DT, ms	205 ± 41	210 ± 39
		*e*′-wave velocity, cm/s	13.5 ± 2.2	14.2 ± 2.1
		*E*/*e*′ ratio	6.28 ± 1.17	6.49 ± 1.23
D’Ascenzi et al. (29)	720 (50% males)	RV basal diameter, mm	40.2 ± 5.1	35.1 ± 4.8
		RV mid-cavity diameter, mm	27.2 ± 4.6	23.9 ± 4.2
		RV longitudinal diameter, mm	89.3 ± 9.5	79.4 ± 9.3
		RV diastolic area, cm^2^	25.1 ± 4.9	19.5 ± 5.2
		RV systolic area, cm^2^	12.2 ± 3.5	9.1 ± 2.7
		RV FAC, %	51.5 ± 8.5	53.3 ± 9.1
		TAPSE, mm	24.3 ± 3.7	23.8 ± 3.3
		*s*′ velocity, cm/s	14.6 ± 2.4	14.1 ± 2.1
Fábián et al. (44)	422 (70% males)	3-D LVEDV index, mL/m^2^	84.3 ± 13.1	74.3 ± 10.5
		3-D LVESV index, mL/m^2^	37.0 ± 7.2	31.3 ± 5.9
		3-D LVSV index, mL/m^2^	47.2 ± 7.6	43.0 ± 6.5
		3-D LVEF, %	56.2 ± 4.0	57.9 ± 4.4
		3-D LV GLS, %	−18.9 ± 2.3	−19.8 ± 2.1
		3-D LV GCS, %	−27.4 ± 2.9	−28.3 ± 3.3
		3-D RVEDV index, mL/m^2^	84.7 ± 14.6	73.6 ± 12.1
		3-D RVESV index, mL/m^2^	38.7 ± 8.8	31.9 ± 7.2
		3-D RVSV index, mL/m^2^	46.1 ± 7.7	41.7 ± 6.7
		3-D RVEF, %	54.6 ± 4.6	56.9 ± 4.8

Data are presented as means ± SD. 3-D, three-dimensional; DT, deceleration time; FAC, fractional area change; GCS, global circumferential strain; GLS, global longitudinal strain; IVRT, isovolumic relaxation time; IVSd, interventricular septum diameter; LV, left ventricle; LVEDD, left ventricular end-diastolic diameter; LVEDV, left ventricular end-diastolic volume; LVEF, left ventricular ejection fraction; LVESV, left ventricular end-systolic volume; LVM, left ventricular mass; LVSV, left ventricular stroke volume; PWd, posterior wall diameter; RV, right ventricle; RVEDV, right ventricular end-diastolic volume; RVEF, right ventricular ejection fraction; RVESV, right ventricular end-systolic volume; RVSV, right ventricular stroke volume; RWT, relative wall thickness; TAPSE, tricuspid annular plane systolic excursion.

**Table 4.** Normative values for biatrial morphology and function in athletes assessed by two- and three-dimensional echocardiography

First Author	No. of Athletes	Parameter	Male Athletes	Female Athletes
Iskander et al. (45)	7,018 (78% males)	LA diameter, mm	36.0 ± 3.2	34.2 ± 3.4
Zaidi et al. (46)	675 (81% males)	RAA, cm^2^	19.5 ± 4.5	17.1 ± 4.0
Roslan et al. (42)	100 (50% males)	LAV index, mL/m^2^	31.1 ± 9.8	28.0 ± 6.2
		RAV index, mL/m^2^	25.2 ± 8.7	19.4 ± 5.3
Lakatos et al. (47)	138 (62% males)	3-D LA total EV index, mL/m^2^	18.0 ± 3.6	19.5 ± 3.7
		3-D LA passive EV index, mL/m^2^	13.7 ± 3.1	15.1 ± 3.4
		3-D LA active EV index, mL/m^2^	4.3 ± 2.1	4.5 ± 2.3
		3-D LA total EF, %	57.2 ± 5.7	59.5 ± 6.7
		3-D LA passive EF, %	44.0 ± 7.4	45.6 ± 7.8
		3-D LA active EF, %	23.2 ± 9.9	25.4 ± 8.7
Lasocka et al. (48)	27 (100% females)	3-D RA total EV index, mL/m^2^	–	16.9 ± 4.7
		3-D RA passive EV index, mL/m^2^	–	10.5 ± 3.2
		3-D RA active EV index, mL/m^2^	–	5.9 ± 2.8
		3-D RA total EF, %	–	53.9 ± 8.3
		3-D RA passive EF, %	–	33.7 ± 9.1
		3-D RA active EF, %	–	27.7 ± 8.6
D’Ascenzi et al. (49)	23 (100% males)	LA PALS, %	39.7 ± 8.4	–
		LA PACS, %	10.6 ± 3.8	–
D’Ascenzi et al. (50)	110 (100% males)	RA PALS, %	40.9 ± 9.9	–
		RA PACS, %	13.1 ± 4.8	–
D’Ascenzi et al. (51)	24 (100% females)	LA PALS, %	–	43.9 ± 9.5
		LA PACS, %	–	15.5 ± 4.0
		RA PALS, %	–	42.8 ± 10.6
		RA PACS, %	–	15.6 ± 5.6

Data are presented as means ± SD. 3-D, three-dimensional; EF, emptying fraction; EV, emptying volume; LA, left atrium; LAV, left atrial volume; PACS, peak atrial contraction strain; PALS, peak atrial longitudinal strain; RA, right atrium; RAA, right atrial area; RAV, right atrial volume.

### Ventricular Morphology

Training has been found to induce ventricular chamber dilatation in both male and female athletes, most notably in endurance counterparts (52). Volume and pressure overload seems to particularly affect the right heart chambers because of their thin walls and greater susceptibility to recurrent increase in pulmonary pressure during exercise (53). Although absolute right ventricular (RV) and LV dimensions were larger in males, after normalization for BSA, females achieved higher values with a larger LV/RV ratio (29). This might be explained by greater LV plasticity and, consequently, greater capacity for cavity enlargement in females, supporting the concept that cardiac remodeling in athletes is a balanced, biventricular phenomenon. Analyzing the thickness of ventricular walls, both RV and LV mass indices were increased in athletes compared with the control group leading a sedentary lifestyle, to a greater extent in males than in females (6, 46). Indeed, as previously mentioned, females adapt primarily by developing eccentric hypertrophy, whereas males show mainly concentric LV remodeling.

**Figure 2.**
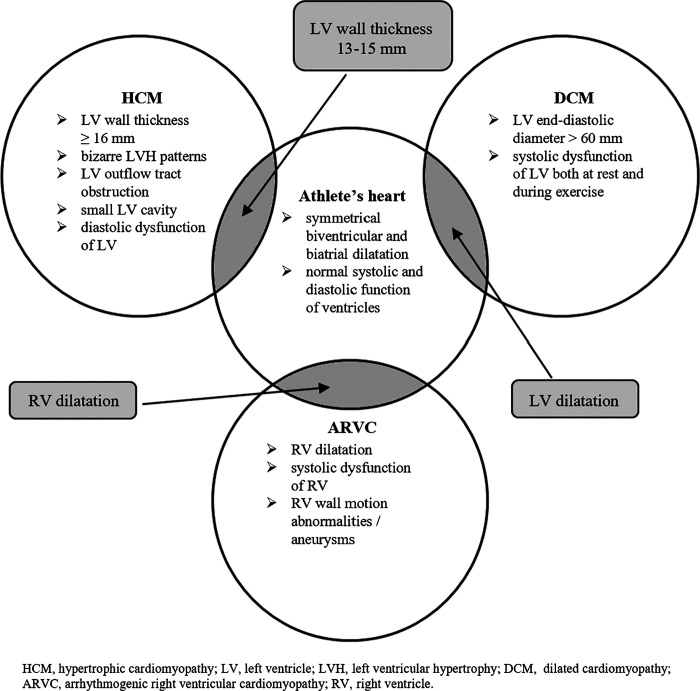
Differentiating echocardiographic features between the athlete’s heart and cardiac disease. The gray zone overlap between physiological remodeling and pathological changes.

In the cohort of 600 elite female athletes, Pelliccia et al. (6) showed that LV end-diastolic diameter exceeded normal limits (>54 mm) in 8% of athletes, whereas only 1% of study counterparts had LV dimensions within the range consistent with primary dilated cardiomyopathy (DCM) (≥60 mm). Maximal LV wall thickness was 14% greater in female athletes compared with sedentary controls, but 23% lower than in male athletes. Importantly, none of female athletes had LV wall thickness in the “gray zone” (between 12 and 16 mm) distinguishing physiological athletic heart from pathologic HCM, in contrast to approximately 2% of male athletes with results ≥13 mm. Furthermore, Zaidi et al. (54) showed that 61% of male and 46% of female athletes exhibit RV dimensions that fulfill the minor criteria for ARVC, whereas 37% of male and 24% of female athletes exceed the major criteria of RV dilatation defining ARVC. Therefore, an enlarged LV raising suspicion of DCM, RV remodeling resembling ARVC or LV wall thickness consistent with HCM is considered more common in male athletes. Such cases should always be thoroughly investigated with the use of various imaging techniques to detect other patterns confirming the diagnosis of underlying cardiovascular disease (Fig. 2).

There are several possible mechanisms—hormonal, molecular, and genetic, underlying the sex-specific alternations in ventricular morphology. Female sex hormone, estrogen, appears to attenuate adverse cardiac remodeling by increasing the expression of the antihypertrophic proteins in the LV, such as myocyte-enriched calcineurin-interacting protein (MCIP1), calcineurin inhibitor responsible for pathological hypertrophy (55). What is more, Pedram et al. (56) observed that treatment with 17-β-estradiol inhibits signaling pathways of angiotensin II and endothelin-1, vasoconstrictors released in response to hemodynamic overload, preventing increase in cardiac mass. In contrast, testosterone induces hypertrophy via androgen receptors’ pathways and cytosolic or nuclear signaling pathways. Androgens activate nuclear factor of activated T cells (NFAT) and inhibit glycogen synthase kinase3 (GSK-3β) leading to cardiomyoblast hyperproliferation (57). Whereas, testosterone deficiency in rats with severe volume overload resulted in reduced LV mass and dilatation, better function, and decreased the expression of genes involved in pathologic LV remodeling (58). Interestingly, several animal studies have shown conflicting results in physiological exercise-induced hypertrophy (59, 60). Female mice exhibited greater hypertrophic response to training than male mice, which might be linked with increased free fatty acid levels and augmented lipolysis, emerging from elevated expression of genes involved in lipid oxidation (60). Indeed, genetic factors are gaining importance, while analyzing cardiac adaptation to training. The angiotensin-converting enzyme (ACE) expression, via kinins degradation and angiotensin II activation, enhances muscle growth and cardiac chamber remodeling (61). In contrast, the administration of an ACE inhibitor prevents cardiac hypertrophy (62). In the study on Japanese endurance runners of both sexes, a significant association between ACE allelic distribution and race distance was detected only in male counterparts (63). However, differences in ACE genotype between sexes and their influence on exercise-induced cardiac changes remain incompletely understood and require further investigation.

### Ventricular Systolic Function

The data available in the literature on the resting systolic function of the athlete’s heart are contradictory. Generally, male and female athletes exhibit both ventricular contractility within the normal range, with no significant differences compared with sedentary individuals (42, 44). However, a recent 3-D echocardiographic study in elite athletes revealed a mild but significant reduction in ejection fraction (EF) of both ventricles, accompanied by a decrease in global strain rate compared with controls (64). The presented outcomes correspond with a recent research on rat models. Sun et al. (65) as first applied the stratified strain technique to determine the left ventricular wall motion abnormalities of each myocardial layer, so as to early detect decreased cardiac function. In response to 8 wk of endurance training, a significant attenuation of LV global longitudinal strain (GLS) and global circumferential strain (GCS) was noticed, following a decreasing trend from the subendocardial layer to the subepicardial layer. The lower systolic values might result from less intense contraction required for larger chambers to maintain stroke volume. The observed reduction in ventricular contractile parameters was more pronounced in male than in female subjects. According to D’Ascenzi et al. (29), who examined 720 Olympic athletes, LVEF and RV fractional area change (FAC) are relatively higher in females, whereas LV and RV tissue Doppler systolic velocities (*s*′) are higher in males. In another study on 163 master athletes, LVEF and RV systolic parameters—FAC and tricuspid annulus plane systolic excursion (TAPSE), did not differ between sexes. Whereas strain rate analysis revealed higher LV GLS and lower LV peak strain dispersion in women than in men (66).

Directly after prolonged endurance training associated with long-term exposure to circulating catecholamines due to desensitization and downregulation of adrenergic receptors, a transient deterioration in LV systolic function was observed (17, 67). A significantly greater decrease in LVEF was found directly after training in males than in females. This discrepancy might be explained by dominant sympathetic regulation both at rest and during exercise in male athletes compared with parasympathetic predominance in females. Consequently, the increased sympathetic response leads to a greater reduction in β-receptor responsiveness and a greater decrease in LV contractility (15, 68). In addition, female sex hormone has a protective effect on the preservation of cardiomyocytes. Although both male and female athletes have estrogen receptors on their cardiac cells, premenopausal women due to increased concentration of the hormone experience greater regulation of genomic and nongenomic cardiac remodeling pathways, greater attenuation of cardiomyocyte apoptosis, greater modulation of LV hypertrophy, and greater adjustment of cardiac contractile function than males (43).

### Ventricular Diastolic Function

Doppler echocardiography in the evaluation of diastolic function in athletes allows to determine the physiological remodeling of the LV and distinguish it from pathologic conditions, such as HCM or myocardial fibrosis, with a pronounced impairment of diastolic performance. In the large cohort of Olympic athletes, Caselli et al. (51) noticed higher early/atrial (*E*/*A*) ratio, mainly due to a decrease in the mean *A*-wave velocity, without significant changes in *E*-wave velocity compared with untrained controls. This means that in athletes most of the LV filling occurs in the early diastole with relatively lower contribution of LA active contraction. All subjects had *E*/*A* ratios > 1.0, with 3% of endurance athletes presenting values >3.0. Furthermore, isovolumic relaxation time (IVRT) and deceleration time of the *E* wave were longer in athletes, whereas early (*e*′) and late (*a*′) diastolic peak tissue velocities were lower in athletes. Finally, *E*/*e*′ ratios, although higher in athletes, were within the normal range in the whole study population, which corresponded with other research (69, 70). Therefore, a restrictive LV filling pattern should only be suspected if there is a marked concomitant increase in the *E*/*A* and *E*/*e*′ ratios.

Taking into account the differences between sexes, it has been shown that female athletes have higher *E*- and *A*-wave velocities than males and, consequently, similar *E*/*A* ratios (71). Although *e*′ and *a*′ velocities were not influenced by sex, *E*/*e*′ ratios were increased in females, presumably as a result of smaller heart size and reduced cardiac volume compensated with elevated filling pressures (66). Therefore, sex-specific alternations in diastolic function seem to be dictated by modifications in LV geometry that are considered more pronounced in males. When analyzing acute changes, directly after prolonged training, a significant reduction in *E*/*A* ratio was noticed, to a larger extent in male than in female athletes (17). Interestingly, a decrease in *E*/*A* ratio following exercise was greater than the concomitant decrease in echocardiographic parameters of the systolic function in both sexes. Among females, differences persist depending on hormonal status. Knebel et al. (72) compared pre- and postmenopausal female athletes and showed a significantly greater reduction in *E*/*A* ratio in postmenopausal runners immediately after the marathon, reflecting a mild diastolic dysfunction, followed by a pronounced elevation in the *E*/*e*′ ratio. However, the observed changes were transient and there were no signs of diastolic dysfunction in the 2-wk observation period.

Echocardiographic evaluation of LV diastolic function, as a noninvasive technique for cardiac passive stiffness prediction, may be used to assess exercise capacity in athletes (73). Cardiac passive stiffness generally refers to the increase in LV end-diastolic pressure, measured indirectly with LV early filling parameters. Molecularly, the giant elastic protein titin is the primary contributor to passive stiffness at physiological muscle sarcomere lengths. Cardiac passive stiffness might be modified by altering titin isoform size or by post-translational modifications (74). In both human (75) and animal models (76), the increased left ventricular passive stiffness correlated with reduced exercise tolerance due to impaired diastolic filling. Similarly, Apor et al. (75) reported that endurance athletes compared with controls have lower resting chamber stiffness and are able to generate a higher cardiac output without increasing filling pressures during exercise. Elaborating sex-specific differences based on the available reports, male athletes have greater estimated wall stiffness and wall stress upon passive filling than females for a comparable amount of training and performance (77), suggesting enhanced exercise capacity in females.

### Atrial Morphology and Function

The remodeling of an athletic heart involves not only the ventricles, but also the atria. With the use of novel echocardiographic techniques, it is possible to characterize atrial morphology and function influenced by training, and to stratify the risk of supraventricular arrhythmias associated with atrial deformation (45). In a meta-analysis on 7,189 athletes, Iskandar et al. (78) showed that the study participants had significantly greater LA size in terms of both LA diameter and LA volume index compared with the control group, to the largest extent in the endurance athletes. According to Mosén et al. (79), who confirmed LA enlargement in male and female athletes, also RA volumes after normalization for BSA were larger in athletes. Moreover, LA and RA response to intensive training is dynamic, and atrial adaptation changes with the exercise loads. Baggish et al. (49) reported an increase in LA volumes in endurance athletes after 90 days of team training. While D’Ascenzi et al. (69) observed a significant biatrial enlargement in a population of female athletes after 16 wk of intensive exercise, but no severe atrial dilatation was noticed in any of the study participants. Taking sex-specific changes into consideration, the aforementioned studies report that both LA and RA volumes are greater in male than in female athletes (78, 79). However, female athletes did not increase their atrial volumes to the same degree as males (79).

To assess atrial function, three phases of atrial contribution to ventricular filling can be assessed: “reservoir” during ventricular systole, “conduit” during early diastole, and “contractile” during late diastole. The soccer player population revealed greater LA reservoir and conduit volumes with stable active volumes compared with the control group. LA reservoir and conduit emptying fractions did not differ between the groups, however, LA active fraction was lower in athletes (71). Recently, speckle tracking echocardiography (STE) became popular in the quantification of atrial myocardial properties. It has been shown that although peak atrial contraction strain (PACS) of the LA was lower in athletes compared with controls, peak atrial longitudinal strain (PALS) of the LA, corresponding with the reservoir phase, remained unchanged (47). On the contrary, 3-D echocardiographic research on 138 elite athletes revealed decreased resting reservoir function of LA, suggesting that LA adaptation to exercise may also involve lower resting reservoir function of the chamber (50). In RA, strain parameters of both reservoir and contractile function were lower in athletes compared with sedentary controls (48). Apart from dynamic changes in atrial morphology, in the study on female amateur athletes we noticed a significant increment in contractile function of LA and RA directly after marathon run (33). Sanz-de la Garza et al. (80) confirmed the postexercise increase in biatrial contraction strains in the population of male runners and showed that the extent of atrial adaptation changes during the training period. Atrial contractile function of both atria increased in distances up to 35 km, with a further decrease post 56 km. Finally, analyzing sex-specific differences, male athletes showed lower values of atrial deformation in the form of reservoir, conduit, and contractile strain rates than female athletes (81). Combining this functional observation with previously reported lower biatrial volumes in women, female athletes are thought to experience less pronounced atrial remodeling than males.

It has been reported that athletes’ atria function at lower strain, larger volumes, and higher wall stress may promote atrial fibrosis and provide a favorable substrate for arrhythmia (82). Recent reports have shown that both atrial enlargement and reduced strain values are strong indicators of atrial fibrillation (AF) (83). Given that the extent of exercise-induced atrial remodeling varies substantially by sex, male athletes are potentially more susceptible to developing AF than female athletes. Nonetheless, the mechanism underlying atrial arrhythmias in athletes deserves further investigation and specific factors, such as age, sex, or sports discipline, influencing onset of AF should be detected.

**Figure 3.**
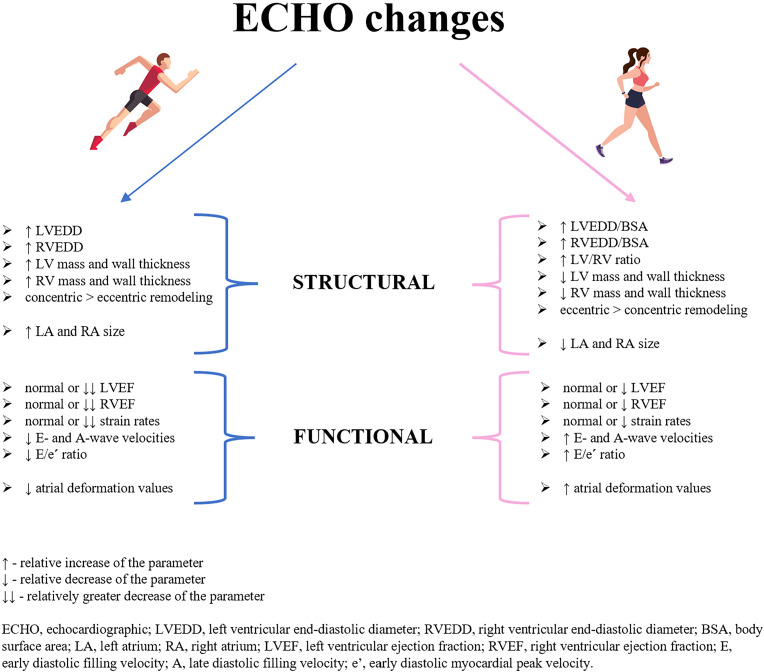
Comparison of echocardiographic changes in male and female athletes.

In conclusion, long-term training promotes the adaptation of all heart chambers with significant sex-specific changes (Fig. 3). Atrial and ventricular enlargement along with decrease of their function may predispose to the development of arrhythmia or be incorrectly interpreted as congenital heart disease. Therefore, it is extremely important to increase healthcare providers’ knowledge on cardiac remodeling in athletes and implement it in the everyday clinical practice.

## CARDIAC BIOMARKERS

**Figure 4.**
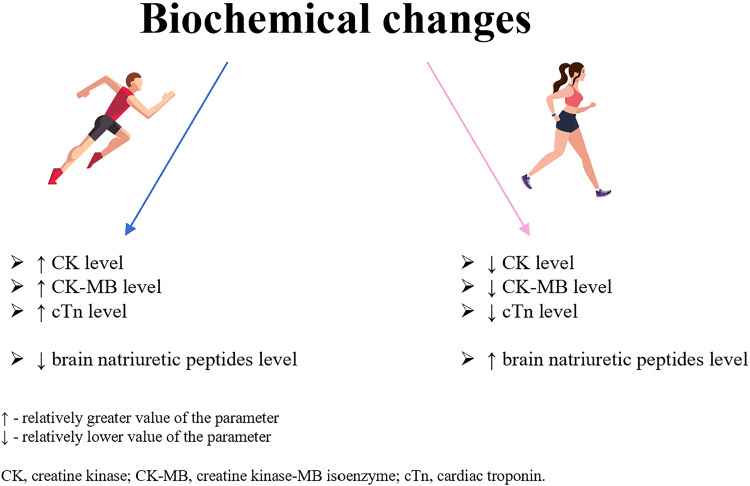
Comparison of changes in cardiac biomarkers in male and female athletes.

Although imaging techniques play the primary role in the evaluation of myocardial morphology and function, the use of cardiac biomarkers should not remain underestimated. Prolonged, intense exercise followed by volume and pressure overload along with neurohumoral activation leads to a transient increase in the concentration of biomarkers of myocardial injury. There are several factors that influence exercise-induced elevation of cardiac biomarkers, including exercise intensity and duration, the athlete’s training experience, baseline biomarker levels, age, and sex. In this review, we will summarize the current knowledge of biomarkers of cardiac injury and overload, including myocardial necrosis enzymes, brain natriuretic peptides, N-terminal proatrial natriuretic peptide (NT-proANP), heart-type fatty acid binding protein (H-FABP), galectin-3 (Gal-3), and proinflammatory cytokines, in a heterogeneous group of athletes, with particular emphasis on sex-related differences in the biochemical response to endurance training (Fig. 4).

### Myocardial Necrosis Enzymes

It has already been reported that prolonged exercise causes a detectable increase in the level of cardiac enzymes, commonly used in screening for myocardial ischemia. Myoglobin and creatine kinase (CK) raised significantly in the vast majority of marathon participants within 1 h after the race and remained elevated for several days post-race (84). This finding may be attributed to continuous regeneration of the skeletal muscle fibers in response to exercise. Apple et al. (85) compared the concentration of creatine kinase-MB (CK-MB) isoenzyme in the serum in subjects after a marathon and after an acute myocardial infarction, showing higher peak CK-MB activity in the runners group than in the patients with acute myocardial infarction. Although CK-MB is known to be primarily active in the myocardium, its levels increase in the skeletal muscle during training and, consequently, the serum CK-MB content also raises following skeletal muscle injury (86). Moreover, athletes participating in daily training have higher resting values of CK than nonathletes, as a cumulative effect of constantly repeated bouts of exercise (87). Mougios (88) introduced even the reference ranges for CK in male and female athletes, which are twice the limits reported for nonathletes. Transient CK increase is considered a physiological reaction to exercise, however, persistence of CK elevation at rest may indicate overtraining or mimic certain pathological conditions, as myopathies (89). When analyzing sex differences in serum CK and CK-MB, male athletes had significantly higher enzyme activities both at rest and after exercise (90). It has been hypothesized that the differences seen between men and women might be explained by variations in muscle fiber recruitment or in muscle mass, however, further research is needed to state the proper underlying mechanism.

Another sensitive and specific marker of myocardial damage is cardiac troponin (cTn). Athletes show higher cTn values at rest compared with sedentary controls (91), which might be explained by heart muscle enlargement caused by repetitive endurance training. The postexercise increase in cTn levels has been reported in various papers on professional and amateur athletes (84, 92–95), and the intensity of training has been found the single strongest predictor of cTn elevation (96). In contrast to acute ischemia, the increase in cardiac troponin levels after endurance training is mild and transient and usually reaches normal values within 24 h (93). In the study on 15 endurance athletes, a persistent minor elevation of cTn was observed in only one subject 5 days after completing a marathon (97). Therefore, the cTn rise should be related rather with cardiac fatigue than actual myocardial damage. However, it cannot be completely excluded that repeated bouts of endurance exercise, each producing subclinical effects, may have a cumulative effect on cardiac structure or function. Studies on animal models provide histological evidence of irreversible myocyte damage corresponding with cTn leakage in response to prolonged exercise bouts (98). So far there is a lack of similar research in humans. Comparing male to female athletes, males have on average higher cTn values than female subjects, both at baseline and postexercise, which might result from previously reported sex-specific steroid hormone discrepancies and, consequently, greater heart mass detected in males than in females (95, 99).

### Brain Natriuretic Peptides

Myocardial wall stress markers, B-type natriuretic peptide (BNP) and inactive N*-*terminal fragment of this pro-hormone (NT*-*proBNP), are released from cardiomyocytes in response to ventricular stretch caused by pressure and volume overload. Hemodynamically stressed heart during endurance exercise can induce transient elevations in BNP and NT-proBNP values (92, 94, 100), probably due to their pleiotropic mechanisms of action. Positive natriuretic effect, vasodilatation, and suppression of sympathetic system evoke preload and afterload reductions and, consequently, decrease myocardial wall tension (93). What is more, in the study on genetically modified mice, the lack of BNP hormone resulted in exaggerated cardiac fibrosis under ventricular pressure overload, indicating antifibrotic force of the factor (101). As under resting conditions, no differences were found in the NT-proBNP levels between endurance athletes and healthy untrained controls, it is hypothesized that repeated bouts of prolonged training do not chronically alter myocardium but represent a physiological adaptation to repetitive hemodynamic loads, and that brain natriuretic peptides might help differentiating physiological and pathological cardiac remodeling to exercise (102). Both male and female athletes present similar reaction of natriuretic peptides, with values within normal limits under resting conditions, and increment after prolonged training (94, 95). However, exercise-induced elevation of natriuretic peptide levels has been shown to be more pronounced in females than in males (103). This may be explained by higher baseline levels of cardiac natriuretic peptides in adult fertile females compared with males of the same age, as various reports indicate that female steroid hormones, in particular estrogens, stimulate the synthesis of cardiac natriuretic peptides (104).

### Other Biomarkers

Apart from the aforementioned cardiac biomarkers, routinely used in daily clinical praxis, there are several novel parameters of growing significance in analyzing cardiac response to endurance exercise. NT-proANP is a marker of atrial wall stretch released in response to increased pressure or heart rate. Acute bout of prolonged training was responsible for significant increase of the marker in healthy marathon runners (105). In addition, higher exercise-induced NT-proANP concentrations positively correlated with an increase in BNP levels, indicating that during intensive exercise both ventricles and atria remain under significant volume and pressure overload (106). Whereas Wilhelm et al. (107) showed that athletes with ≥5 marathon participations had significantly larger atria and higher baseline levels of NT-proANP compared with less experienced marathon runners and control subjects, which might be explained by repetitive episodes of atrial stretching causing persistent atrial remodeling. Similarly, as for brain natriuretic peptides, females have on average higher NT-proANP values than males due to sex steroid hormone discrepancies (108). In animal model, estrogens were reported to increase *ANP* gene expression in a dose-dependent manner and to maintain suitable levels of *ANP* gene expression in rat cardiomyocytes, whereas androgens are suspected of inhibitory effect on ANP secretion in atria (109). However, there lack of specific data on differences in NT-proANP levels between male and female athletes.

Furthermore, biomarkers of transient myocardial injury include growth and differentiation factor 15 (GDF-15) and H-FABP. Although these parameters are not pathognomonic for cardiomyocyte damage, their increased levels were shown to predict outcomes in patients with coronary artery disease or heart failure (110). Acute hemodynamic changes during endurance training promote elevation of both GDF-15 and H-FABP values, which return to normal ranges within 72-h post-exercise and, therefore, should be regarded rather as a cytoprotective and growth-regulating effect than a pathological reaction (106, 111). Although in animal models female rats had significantly greater H-FABP concentrations than males due to estrogen-dependent increased lipid oxidation (112), the corresponding research in humans is scarce. Consequently, the sex-specific response to training of the discussed parameters is unknown.

Inflammatory response to excessive training results in fibroblast proliferation, collagen production, and myocardial fibrosis (113). Gal-3 and suppression of tumorigenicity 2 (ST2) represent markers of cardiac remodeling and fibrosis. Gal-3 is a lectin family protein released by activated macrophages, whereas ST2 belongs to the interleukin 1 (IL-1) receptor family induced by increased cardiac wall tension. Both parameters predict mortality in patients with acute and chronic heart failure. Le Goff et al. (114) showed that Gal-3 and ST2 levels increase postexercise, to the largest extent in the group of endurance athletes, i.e., marathon runners. Besides, repetitive training may cause morphological and functional changes in the myocardium leading to higher baseline values of presented biomarkers than detected in nonathlete controls (113). Although the knowledge on novel biomarkers and their use in monitoring of exercise-induced cardiac remodeling is constantly growing, the vast majority of available reports concern male athletes (115, 116). Further research is needed to better illustrate sex-specific changes.

## CONCLUSIONS AND CLINICAL IMPLICATIONS

Exercise leads to electrical, structural, and functional changes in the cardiovascular system, also known as “athlete’s heart,” with endurance sports being associated with the greatest degree of cardiac remodeling. Most of the available research on cardiac adaptation to training is derived from data collected in men. However, as the number of females participating in various sport events is constantly growing, more complete understanding of physiological characteristics and limits in women is a priority scientific task. Therefore, we decided to summarize the existing differences in cardiac response to training between male and female athletes, focusing on underlying physiological mechanisms that determine sex-specific reaction and potential clinical implications.

Although the geometry of the heart remains unaltered in the majority of subjects, repeated bouts of endurance exercise, associated with an increase in preload and afterload, induce cardiac remodeling with significant differences between sexes. As presented in the review, athletes have on average greater biventricular wall thickness and dimensions with concomitant mild reduction in diastolic and systolic function, compared with untrained controls. Males present rather with concentric hypertrophy, whereas eccentric remodeling dominates in females. The observed changes in ventricles correspond with atrial dilatation and decreased functional parameters, to a greater extent in male than in female athletes. The signs of an overloaded heart include also larger concentrations of cardiac biomarkers released from damaged myocytes in response to recurrent increases in volumes and pressures. Apart from chronic adaptation to endurance exercise, acute bouts of training load result in comparable structural, functional, and biochemical alternations in the cardiovascular system, more pronounced in the amateur less-trained subjects than elite athletes with daily practice routine. However, these changes are transient and should resolve after detraining period.

Recognizing physiological cardiac response to training in males and females is of clinical importance, as certain phenotypes may mimic inherited pathological conditions. Dilated LV with low-normal or mildly reduced systolic function raising suspicion of DCM, RV enlargement resembling ARVC or LV wall thickness consistent with HCM should always be thoroughly investigated to detect other patterns confirming the diagnosis of underlying structural heart diseases. Moreover, both atrial and ventricular adaptation to training in the form of enlargement and reduction in function predispose to the development of arrhythmias. As the observed changes are considered more pronounced in male athletes, it is not surprising that the risk of supraventricular and ventricular heart rhythm disturbances and, consequently, the incidence of SCD is higher in males than in females. In addition, unexplained cardiac deaths during exertion might occur in individuals with morphologically normal hearts and primary electric diseases, such as ion-channelopathies or Wolff–Parkinson–White syndrome, defined by specific ECG patterns. The question arises whether all athletes should be monitored for pathological cardiac adaptation or just individuals with genetic predisposition and the positive family history of inherited heart disease. To what extent can the observed abnormalities be caused by repeated bouts of intense exercise and be reversed with suspension of training? Furthermore, should all or only selected athletes with the most profound remodeling be screened for potential arrhythmias? Finally, considering cardioprotective role of estrogen and its positive influence on metabolic processes and gene expression, are female athletes in fact less susceptible to develop pathological remodeling than males? Are there any other physiological mechanisms, apart from those discussed in the review, potentially responsible for sex-specific cardiac response?

Based on the available knowledge from various scientific sources, we would recommend cardiovascular screening in the form of physical examination with anthropometric data, ECG, echocardiographic assessment, and basic biochemical blood tests in elite and amateur athletes of both sexes. Combination of the results of these noninvasive examinations would increase the probability of proper distinction between physiological cardiac adaptation and pathological remodeling. Moreover, it would help to identify subjects requiring further evaluation, such as cardiac magnetic resonance imaging or Holter ECG monitoring. Acute changes in cardiac biomarkers’ levels could also be applied by trainers to control and modify the intensity of training program, based on cardiovascular reaction to various exercise loads. We hope that data on specific alternations in athlete’s heart provided in this review would increase the healthcare providers’ awareness of exercise-induced cardiac remodeling and induce greater implementation of available screening techniques in the everyday clinical practice.

## DATA AVAILABILITY

Data will be made available upon a reasonable request to the corresponding author.

## DISCLOSURES

No conflicts of interest, financial or otherwise, are declared by the authors.

## AUTHOR CONTRIBUTIONS

Z.L.-K. and A.D.-K. conceived and designed research; Z.L.-K., Z.L.-P., A.K.-D., A.S., L.K., E.L. and A.D.-K. analyzed data; Z.L.-K. prepared figures; Z.L.-K. drafted manuscript; L.K., E.L., and A.D.-K. edited and revised manuscript; A.D.-K. approved final version of manuscript.
